# Being at Peace as an Important Factor in Acquiring Teaching Competency by Iranian Nurse Teachers: A Qualitative Study

**DOI:** 10.5539/gjhs.v6n3p109

**Published:** 2014-02-24

**Authors:** Hormat Sadat Emamzadeh ghasemi, Forough Rafii, Mansoureh A. Farahani, Nooreddin Mohammadi

**Affiliations:** 1 Schools of Nursing and Midwifery, Iran University of Medical Sciences, Tehran, Iran; 2 Department of Nursing Management, Schools of Nursing and Midwifery, Tehran University of Medical Sciences, Tehran, Iran; 3 Center for Nursing Care Research, School of Nursing and Midwifery, Iran University of Medical Sciences, Tehran, Iran

**Keywords:** teaching competency, nurse teachers, grounded theory, acquisition competency

## Abstract

It is imperative to understand the factor that influence teaching competency. Therefore, it is necessary to study those that have an impact on the process of acquiring teaching competency. Competent nurse teachers have an important role in the achievement of nursing students and improving the quality of nursing education. However, few researches have focused specifically on the process of acquiring teaching competency in nurse teachers and its related factors. This study as a part of more extensive research aims to explore the factors influencing acquisition of teaching competency by Iranian nurse teachers. Grounded theory was chosen as the method. Eleven teachers from three nursing schools in Tehran were recruited. Data was generated by semi structured interviews during May 2011 to March 2013 and was analyzed through using constant comparison. Three main categories were emerged including “individual characteristics” (spirituality, professional interest, ethical conducts, knowledge expansion and reflective practice), “organizational factors” (management of educational systems, solidarity culture, student characteristics) and “socio-cultural factors” (social situations, and public definition of nursing). Nurse teachers who deal peacefully with the nursing profession and colleagues are responsible and committed to acquiring teaching competency. A suitable organization in nursing educational systems that is structured and ordered also encourages a peaceful approach by nurse teachers.

## 1. Introduction

Prosperity toward superior nursing care quality within the complex and ever-changing health care and higher education environment requires nurse teachers with adequate knowledge, skills and abilities (Johnson-Farmer & Frenn, 2009; Thornlow & McGuinn, 2010). It is obvious that if the nurse teachers want to be successful in their profession, they need to achieve competency in nursing education. Teaching competency is required to train students who are capable and have a positive perspective to care (Holopainena, Hakulinen-Viitanenb, & Tossavainen, 2007; Johnson-Farmer & Frenn, 2009).

Teaching competency is the ability to meet the needs and requirements of the teaching profession, which manifests in teachers’ actions and reactions as they apply an integrated set of knowledge, skills, and attitudes (Nijveldt, Beijaard, Brekelmans, Verloop, & Wubbels, 2005). Competency has been called as “knowing in action”, that is an automatic response to the present condition based on internalized knowledge, which increases during a teacher’s tenure (Lysaght & Altschuld, 2000).

Acquiring teaching competency by nurse teachers depends on their attitude and their profession’s representative way of working, as well as the individual’s interpersonal skills and characteristics (Holopainen, Hakulinen-Viitanen, & Tossavainen, 2007; Johnson-Farmer & Frenn, 2009). It requires the establishment of an interpersonal relationship that is influenced by different social conditions and contexts (Holopainen et al., 2007; Holopainen, Tossavainen, & Kärnä-Lin, 2008). Therefore, becoming a competent nursing teacher is a multidimensional and dynamic process that is influenced by factors such as changing process in the organisation, the culture of health care, nurse teachers’ professional self-esteem, the focus of nurse teachers’ competence, their relationship with students, the future of their profession, and requirements for staying in the profession (Holopainen et al., 2008).

In the last couple of decades, many researchers have studied the action of nurse teachers and challenges to their competence throughout the world. However, more studies need to be conducted in this area because there are different expectations among societies in relation to professional competency and situations (Holopainen et al., 2007; Johnsen, Aasgaard, Wahl, & Salminen, 2002). In addition, the concept of teaching competency in nursing has cultural complexities that result from changes in societies, scientific and technical advances in nursing, experiences gained in the job, and the culture and working conditions in different countries (Holopainen et al., 2008; JafariGolestan, Vanaki, & Memarian, 2007).

It should be considered that the mission and primary goal of nursing schools is to train competent nursing student and thus to provide quality nursing services to patients (Johnson-Farmer & Frenn, 2009; Little & Milliken, 2007; Thornlow & McGuinn, 2010; Vanaki & Memarian, 2009), which it is not possible unless the nursing schools are equipped with competent nurse teachers. Therefore, deep knowledge of the factors influencing teaching competency by nurse teachers in every cultural and social context can help to overcome obstacles and supports the facilitators to design effective staff development programs for nurse teachers.

### 1.1 Background on Nurse Teachers in Iran

Most of the Iranian nurse teachers have a Master’s degree (MSN) or PhD in nursing and are recruited based on their desire to work in nursing faculties. Most of the recruitments are based on the civil service system in Iran. Some of the nurse teachers, however, work part time who are employed without consideration of any criteria of teaching competency. Nurse teachers in Iran promote and achieve some degrees of professional competency by participation in staff development programs, doing research projects, publishing papers in nursing journals, and attending national or international conferences or workshops.

### 1.2 Aim

The aim of this study was to explore and describe the factors influencing the acquisition of teaching competency by Iranian nurse teachers

## 2. Method

This report is part of a doctoral dissertation aimed at shedding light on the process of acquiring teaching competency by nurse teachers. Gaining teaching competency is a dynamic process that occurs in a variety of socio-cultural contexts. Grounded theory is rooted in symbolic interactions (Jeon, 2004), and is sets out by researchers to discover patterns of behaviour among particular groups of people in specific contexts (Backman & Kyngäs, 1999). So grounded theory approach was used for data collection and analysis in this study (Corbin & Strauss, 2008), which is a suitable method to reach a deep and multidimensional understanding about acquiring teaching competency and its influential factors.

### 2.1 Participants and Data Collection

Data collection was started in May, 2011 and continued until March, 2013. Purposive sampling was used for selecting participants (Patton, 2002), which selecting criteria were that participants should be recognize as competent nurse teacher by peers, students, and heads of department. Theoretical sampling was used to maximize the opportunities to develop concepts and emerging categories (Corbin & Strauss, 2008). Therefore, data was collected by semi-structured interviews with competent nurse teachers in primary interviews and continued with theoretical sampling from others nurse teachers in subsequent interviews (n=11). Some of the interviews were carried out more than once to obtain further explanations or clarifications of certain statements (n=16). Saturation was reached after 10 interviews and analyses from 7 participants, which four additional participants were sampled to ensure the quality of the information. All of the participants were full time nurse teachers from three nursing schools of Tehran who two participants had masters’ degree and nine had PhD in nursing education. The PhD nurse teachers consist of: Sex competent nurse teachers, two member of Iranian board of nursing and one was nurse teacher with more than 30 years experience in nursing education. The range of work experiences of them was 5 - 31 years (mean: 25.3 years). The interviews were carried out in the participants’ offices and the correspondent author conducted all the interviews, which were guided individually and lasted between 30 minutes and 1.35 hours (mean: 1.0 hour).

Interview guide consisted of grand open ended questions to allow the respondents to explain their experiences as complete as possible. The participants were asked to explain their own experiences and perceptions about teaching competency in nursing, and the situations which facilitate or hinter their teaching competency acquisition and also their strategies to overcome the barriers. For example: Please tell me about your teaching sessions in the school of nursing, Tell me about the first/last days of teaching, What is your perception of teaching competency in nursing? Will you please tell me, which activities you usually doing to acquiring competency? How have you acquired teaching competency in nursing? What factors have been effective on teaching competency acquisition?

Probing questions were also used to clarify information and gain additional data. Participant recruitment, data collection, and data analysis continued until theoretical saturation was reached and a rich description of experiences was obtained. All interviews were tape-recorded and, transcribed verbatim and then analyzed with using constant comparative method (Corbin & Strauss, 2008). Data collection and analysis took place concurrently.

### 2.2 Data Analysis

Data analysis was carried out in four main phases. In the first phase, the data was examined for concepts, deriving codes from the interview data and distinguishing theory categories and their properties. Then, elaborate analysis was conducted by connecting the concepts to each other, crosscutting, comparing incident for similarities and differences, and theoretical sampling. In the next phase, which was done concurrently with the mentioned phases, the data was analyzed for context and identified sets of conditions that influence on acquiring teaching competency of nurse teachers. In the third phase, we identified strategies used by nurse teachers to deal the problems they face in process of acquiring teaching competency. The fourth and final phase focused on detecting core categories. Writing memos and story lines were used to cross-examine the data throughout the analysis. MAXQDA 2010 software was used to manage data.

### 2.3 Methodological Considerations

This study evaluated in terms of trustworthiness, credibility and dependability (Corbin & Strauss, 2008; Lincoln & Guba, 1985). Trustworthiness was used to make certain integrity and truthfulness from the start of the research process. In order to present a summary of the transcribed interviews with the initial codes were provided to the most of the participants as member checking to confirm that the researchers had real understanding from their world. In addition, a continuous comparison between data, codes and categories was made throughout the analysis. Credibility was established through the participants’ confirmation of our understanding by member check, prolonged engagement with the field, peer check, external check and, maximum variation of sampling, also by presenting views from different participants who were purposely and theoretically chosen (Corbin & Strauss, 2008; Streubert & Carpenter, 2010). Dependability was assured by the fact that the same researcher, the correspondent author, performed all the interviews, analysis and transcriptions. The use of a tape-recorder and verbatim transcripts, as well as referring back to, and re-reading the transcripts during the analysis process, allowed the researcher to stay near to the text. The citations make it possible to assure conformability. Objectivity of the data was enhanced by ensuring theoretical sensitivity whereby the researcher put aside any preconceived ideas about the topic during the data analysis. Then, expert supervisors and faculty members checked the data and its objectivity.

### 2.4 Ethical Consideration

The Ethics Committee of Tehran University of Medical Sciences approved this research. All participants provided informed consent. We assured the participants of their anonymity and informed them that they could stop the interview at any time they wished. The researcher also obtained the participants’ permission to audiotape the interviews to study and audit trail.

## 3. Findings

**Figure 1:**
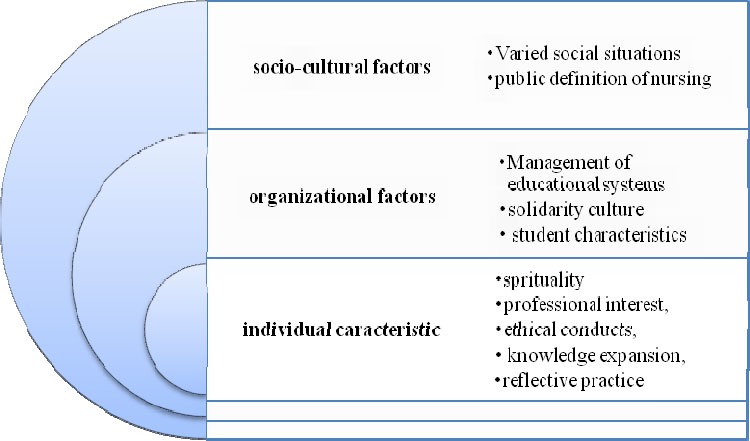
The factors influencing on teaching competency in the participants

The findings showed that three main categories of “individual characteristics”, “organizational factors and “socio-cultural factors” have influenced teaching competency in nurse teachers. Figure 1 depicts the emerged categories and their sub-categories.

### 3.1 Individual Characteristics

Individual characteristic as an intrinsic factor influenced teaching competency process in nurse teachers. This category consisted of five subcategories: “spirituality”, “professional interest”, “ethical conducts”, “knowledge expansion” and “reflective practice”.

#### 3.1.1 Spirituality

For participants in this study, spirituality provided a sense of satisfaction and efficacy throughout the process of acquiring teaching competency. They tried to achieve spiritual goals and along with that came a sense of commitment and job accountability that encouraged them to improve their teaching competency. One of the competent nurse teachers stated (P_3_): “*I always ask God to help me to develop my teaching and learning skills during the teaching process; this consideration has been very helpful, but whenever I neglect God or relay just on myself the learning results have been very poor”*.

Also, a sense of accountable to God led nurse teachers to be at peace with living and people that augmented the nurse teachers’ capability and professional competency. One of the competent nurse teachers stated (P_2_): *“We have to be accountable somewhere, the first is God, and God tells us help my people”*.

#### 3.1.2 Professional Interest

For participants in this study, teaching competency depended on the level of interest in nursing and caring. They believed that different attitudes toward nursing education not only influence teachers’ competency and ability, but also affected students’ competency. One of the competent nurse teachers, stated (P_4_): *“Thence, I accept nursing profession and adapt with nursing…so, my standpoint was always that our outcome must be for the patient”*. On the contrary, performance of the teachers with negative views about caring and nursing, effect as a barrier on the teaching competency process. In this regard, the other competent nurse teachers (P_5_) quoted from some incompetent of her colleagues: *“Caring for patients and helping them is regarded as a degrading action for nursing students and nurse teachers”*, also she stated: “*These teachers tell me that you lower their prestige”*. Hence, the different and opposing perspectives of nurse teachers and different levels of interest in nursing and caring influenced their teaching competency.

#### 3.1.3 Ethical Conduct

The participants believed that their professional ethics resulted in the development of students, the progress of their profession, and their own competency. The ethical behavior of the teachers establishes good communication with their group manager and colleagues. For instance, the teachers’ commitment to performing the duties perfectly and solving the existing problems resulted in their greater efforts for the acquisition of teaching competency. One of the competent nurse teachers stated (P_1_): *“I can work properly with head nurses in clinical settings; also, I can get along well with the group manager. For example, very often, the group manager asks me to not take this or that lesson because teacher X is taking it. I simply get along with her”*. In this regard another competent nurse teacher stated (P_7_): “*When we become seniors (in nursing education) as some competencies, we should help juniors’ promotion and this very thing conveys more energy to us to move forward and become more competent”*. So being at peace with others and loving them will result in improving teaching competency in nurse teachers.

#### 3.1.4 Knowledge Expansion

For the participants, knowledge expansion was one of the effective teacher’s characteristics in the process of achieving teaching competency. The participants declared that in order to develop their knowledge, they did self-study, participated in scientific conferences, attended in clinical settings, and also in other teachers’ classes. In this regard, one of the nurse teachers stated (P_3_): “*I try to study before going to class and review the content which I want to teach, because I believe that my pervious knowledge is not enough”*. Another teacher stated (P_6_): *“I had just finished my Master’s degree and I had to be a nurse teacher for students in critical care unit. So, I myself went to the unit in the afternoons to learn more”*.

#### 3.1.5 Reflective Practice

By working more and studying harder, learning from previous experiences, and reflection on what they had learned, the participants consistently took measures to improve their teaching quality. In this regard one of the competent nurse teachers stated (P_1_): “*I thought a lot on a series of teaching principles which I had learnt in the teaching courses”*. Another participant stated (P_7_): *“We should recall our previous experiences and try to learn from the events that happened to us”*.

### 3.2 Organizational Factors

This category consisted of three subcategories including “management of educational systems”, “solidarity culture”, and “student characteristics”.

#### 3.2.1 Management of Educational Systems

From the teachers’ views, management of educational systems in terms of decision making, implementation of rules and regulations, staff development and evaluation programs should be in such a way to provide the teachers with a sense of security and peace in the job environment or a sense of disappointment. One of the competent nurse teachers stated (P_4_):*“When you see that the system is not the one you can exercise your thoughts and ideals, you’d rather give up and stop”*. Also, the promotion of teachers and evaluation of their performance based on quantitative rather than qualitative criteria played an inhibitive role in the process of acquisition competency in nurse teachers. In this regards, one of the competent nurse teachers stated (P_1_): “*New rules limit us more each day. They say you have to have two papers indexed in ISI. It preoccupies the teachers or makes them put force on students to write these papers or they do it themselves, because this is what brings promotion, not a good class”*.

#### 3.2.2. Solidarity Culture

Existence or lack of solidarity among colleagues was a subcategory of organizational factors which affected on acquiring teaching competency. As a result, encouragement and persuasion of peers and providing sincere and respectful feedback to each other facilitated the process of teaching competency acquisition. As an example, a competent nurse teacher said (P_1_): “*In the first session of my class, Dr. X came with me and after the class said that I taught very well, he encouraged me very much and I taught the next session more comfortably and more easily because of his encouragement. … My colleague was an important factor in my improvement”*.

#### 3.2.3 Students’ Characteristics

Since effective teaching was associated with the students’ motivation and their tendency to learn, teachers had different educational conducts and motivations. They performed different activities to achieve competency which were proportionate to different behaviors and feelings of their students (P1): *“I feel a severe decline…I think with myself that students are no longer interested in science, they don’t like to learn”*. And another participate stated (P_3_): “*My satisfaction and success is different at different periods, because students themselves are different”*.

### 3.3 Socio-Cultural Factors

This category consisted of two subcategories including “various social situations” and “public definition of nursing”.

#### 3.3.1 Various Social Situations

The teachers who encountered various situations in their work and society could attain teaching competency easier than others: “*I dealt with different fields. I learnt some things from the pharmacist, some things from the psychiatrist, etc. Each one somehow influenced me”*. Different situations and opportunities that nurse teachers encountered in their profession made them more competent than their counterparts.

#### 3.3.2 Public definition of nursing

Public definition of nursing profession and the social stand of nurses provided various levels of motivations for the teachers to acquire competency: “*Professional anonymity in the society is a barrier…in addition, lack of a specific definition of nursing and its power in health systems are other barriers. These barriers are challenging for us and inhibit us to accept the profession of nursing and get competency”*.

## 4. Discussion

The findings of this study indicated that teaching competency for nurse teachers was influenced by individual characteristics of the nurse teachers, organizational factors in nursing educational systems, and socio-cultural factors in their living and background. In addition, the findings indicated that the participants’ characteristics and their interest about nursing profession and colleagues had an important role in acquiring teaching competency and confronting the existing challenges in the way of acquiring teaching competency. In a qualitative study by Holopainen et al., teaching in nursing profession was introduced as a multidimensional and dynamic process, which was influenced by factors such as the process of organizational changes, the culture of governing health care and social systems, the teachers’ professional self-confidence, their interaction with students, their image of their future occupation, and their personal needs and characteristics (Holopainen et al., 2008; Holopainen, Tossavainen, & Kärnä-Lin, 2009). The findings of our study also showed that most of these factors such as professional characteristic of nurse teachers, peaceful interactions of teachers (as a cultural value), and the culture of their organizations and managerial systems had the greatest effect on their teaching competency. Consequently, the nurse teachers, based on their individual characteristics and their attitudes towards nursing and teaching, tried to respect the cultural values and acquire teaching competency.

The nurse teachers, according to their views and acceptance of nursing profession, performed different activities in the teaching career. Some of them who had positive views about the concept of nursing and nursing teaching dealt with peacefully and always tried to develop nursing capabilities in their students and their selves. Also, they tried to solve problems in clinical nursing and nursing education. Likewise, Holopainen et al. (2009) indicated that the commitment of nurse teachers was as a main factor of nurse teacherhood and played a pivotal role in their teaching activities. Therefore, it can develop different capabilities for these teachers based on the levels of commitment to the teaching profession (Holopainen et al., 2009). In our study, the teachers who had interest in and commitment to nursing profession tried to have a complete understanding of the educational contents, to transfer knowledge to others better, and to facilitate the process of education. In this process, the nurse teachers, had a feeling of commitment to the nursing profession, tried to develop competency and empowerment in their students, and solve the existing problems in the system. Moreover, such endeavors resulted in an increased professional competency of the teachers.

Johnson et al. (2009) described that the process of becoming an excellent nurse teacher was changing from ‘instiller’ to ‘facilitator’ and laid the foundation for continued development of our teaching in nursing (Johnson-Farmer & Frenn, 2009). Therefore, it can be argued that by fostering the facilitative role of the nurse teachers, it will be possible not only to achieve success in effective teaching to nursing students, but also to further foster the teachers’ capabilities and competency. According to the findings of the present study, by facilitating the students’ learning and increasing their capabilities, the competent teachers themselves acquired more capabilities and competency based on the principle of “reciprocal effect of functions”: *“This world is the mountain and our action the shout: the echo of the shouts comes (back) to us” (Maulana Rumi, 1925)*.

In addition, the findings of the present study showed that nurse teachers with lower degrees of commitment and interest in nursing profession not only place in lower levels of nursing teaching competency, but also do not possess the necessary job satisfaction from performing nursing activities and teaching its specific skills and features. Such teachers, as stated by the participants, were regarded as barriers to the acquisition of teaching competency for other teachers. In general, teaching and nursing education are dependent on a representative way of professional working, interpersonal skills, and the teacher’s personal characteristics (Holopainen et al., 2007). If the interpersonal skills and relationships of nurse teachers in the work environment are not for the actualization of supreme organizational goals, they can be considered as a barrier to the acquisition of job competency and empowerment for nurse teachers.

It is obvious that, since teachers need to establish interpersonal relationships, they are influenced by different social situations and contexts (Holopainen et al., 2007). The findings of our study showed that the existence or absence of solidarity among colleagues, as a subcategory of organizational factors, influenced teaching competency acquisition. Peers’ encouragement and persuasion long with providing sincere and respectful feedbacks facilitated the process of teaching competency acquisition. In this regard, is believed that the interface between practice and theory of ethics should be unifying by the harmony created by being at peace with one’s cultural values (Mpeli & Monnapula-Mapesela, 2009). In our study, the teachers were at peace with their colleagues and their cultural organization values; they tried to develop unity, create a spirit of cooperation in the work environment, and receive more help and cooperation from their colleagues. These teachers possessed ethical values and professional ethics, made provisions for acquiring teaching competency.

It has been argued that the individual characteristics of teachers are as influential factors in nursing education (Najafi Kalyani, Sharif, Nahid, & Shahnaz, 2011). Price (2010) also regarded the personal factors in teachers as a determinant of their reactions to their work load in colleges and their abilities in reaction to work environment stresses. The findings of our study also indicated that the individual factors of teachers were one of the three major categories of influential factors in teaching competency acquisition. According to other studies, this factor is necessary for a nurse teacher to enter the field of education and to promote these competencies in clinical setting (Johnson-Farmer & Frenn, 2009; McAllister & McKinnon, 2009).

The participants had different behaviors based on their own commitment and perspectives towards the nursing profession and nursing education. Therefore, they experienced different levels of empowerment and satisfaction which finally resulted in different educational outcomes. The findings of the present study showed that the nurse teachers’ views about the nursing profession and their relationships with colleagues were important elements of individual characteristic. Nurse teachers with dealing peacefully toward nursing profession and colleagues are responsible and committed to acquiring teaching competency and overcoming the obstacles in process of teaching competency.

## 5. Conclusion

Teachers’ activities in the acquisition of competency were according to their attitudes towards nursing profession, colleagues, and all other people. Therefore, the process of acquiring competency by nurse teachers is situated on a spectrum with complete teaching competency in nursing on one end, and incompetency on the other end. However, it is difficult to place nurse teachers in one of the extremes because of the dynamic nature of human beings, education, and nursing profession. In addition, different levels of “being at peace” with profession, colleagues and people would result in different levels of teaching competency for the nurse teachers. They are always in the process of development and dynamism, based on their views about and definition of nursing, humanity, and commitment. Hence, they reveal different behaviors and reactions to the existing challenges in the process of competency acquisition. In other words, despite various intrinsic and extrinsic factors that could act as barriers or facilitators in the way of competency acquisition, if nurse teachers possess individual characteristics with positive attitudes and dealing with peacefully could easily pass the process of teaching competency acquisition with success and move towards supremacy.
